# Meniscus Tear Morphology and Patient Demographics as Predictors of Treatment for Meniscal Tears: A Natural Language Processing Study

**DOI:** 10.1177/23259671251397648

**Published:** 2026-03-20

**Authors:** Drew A. Lansdown, Kian Niknam, Madeleine Orringer, Jason Crane, Carolina Ramirez, Thomas M. Link, Sharmila Majumdar

**Affiliations:** † Department of Orthopedic Surgery; Sports Medicine & Shoulder Surgery, University of California, San Francisco, San Francisco, California, USA; ‡ Department of Surgery, New York University Langone Hospital–Long Island, New York, New York, USA; § Department of Orthopedic Surgery, University of Southern California, Los Angeles, California, USA; ‖ Department of Radiology & Biomedical Imaging, University of California, San Francisco, San Francisco, California, USA; Investigation performed at the University of California, San Francisco, San Francisco, California, USA

**Keywords:** meniscus tear, knee osteoarthritis, knee MRI, natural language processing.

## Abstract

**Background:**

Meniscus tears occur in tandem with other degenerative changes at the knee joint, making interpretation of imaging findings challenging. Meniscus tears, commonly treated by general physicians and musculoskeletal specialists, have significant treatment variability.

**Purpose/Hypothesis:**

The purpose of this study was to investigate whether big data analytical tools and natural language processing (NLP) of magnetic resonance imaging reports could identify factors that are predictive of treatment decisions in a large cohort of patients. It was hypothesized that surgical treatment would be associated with specific meniscus tear patterns.

**Study Design:**

Cross-sectional study; Level of evidence, 3.

**Methods:**

Deidentified electronic health records from approximately 5 million patients, available at Information Commons, were analyzed to identify patients with meniscus tears on knee MRI reports. NLP was used to extract descriptive features from MRI reports. Demographics and clinical and treatment data were recorded. Univariate and multivariate analyses were performed.

**Results:**

For 4013 patients with meniscus tears on MRI, physical therapy was the most common treatment (62.4% of patients). Opioid medications were prescribed to 25.6% of patients diagnosed with a meniscus tear. Surgery was performed for 19.6% of patients. A bucket-handle tear was the strongest predictor for receiving surgery. Meniscus tear patterns, the presence of degenerative changes, and demographic factors were independent predictors of treatment received.

**Conclusion:**

Meniscus tear types and other findings at the knee joint, along with demographic variables, are associated with treatment for meniscus tears. Bucket-handle and root tears were most likely to undergo surgical treatment, while patients on Medicare and those with arthrosis were more likely to be treated with nonoperative modalities. NLP of big data, including radiology findings, may have a role in providing clinical decision support for interpreting findings on knee MRIs. Prescription of opioid medications in meniscal tears was observed at a higher-than-expected rate.

Meniscus tears are one of the most common knee injuries treated by general physicians and musculoskeletal specialists.^18^ The meniscus is a fibrocartilaginous structure that serves an important role in distributing load across the tibiofemoral joint.^2^ Tearing of the meniscus may occur as a result of an acute injury or in the setting of osteoarthritis and degenerative joint disease.^8^ The diagnosis of a symptomatic meniscus tear remains difficult, as physical examination tests and symptoms are often nonspecific.^24^ Meniscus tears are often grouped together as 1 entity, although different tear patterns can produce very different effects on knee function. Some tear patterns, such as meniscus root tears and radial tears, result in complete disruption of meniscus function, while others, like horizontal tears, may have less of an effect on knee kinematics.^2^

Treatment for meniscus tears remains controversial, and the patient-specific factors that drive treatment decisions remain unclear.^25^ Some symptomatic meniscus tears may be managed successfully with some combination of oral medications, intra-articular injections, strengthening exercises, and activity modification, although arthroscopic meniscus surgery remains one of the most common knee surgeries performed in the United States.^13,27^ Outcomes after partial meniscectomy surgery in middle-aged patients with varying degrees of arthritis are generally no different in aggregate than outcomes for patients treated nonoperatively.^11,12,14^ Conversely, specific tear types, such as bucket-handle tears and meniscus root tears, benefit from arthroscopic surgical management, with superior outcomes achieved through meniscus repair surgery.^1,3,4,9,15,16,28^

Magnetic resonance imaging (MRI) remains the primary imaging modality for diagnosing meniscus tears, although there is a high prevalence of meniscus tears on MRI, even in the asymptomatic knee of the middle-aged or elderly patient.^7^ Knee-related symptoms can be from tearing at the meniscus itself or from a range of other morphologic changes at the knee.^5^ Meniscus tears occur regularly with other degenerative findings at the knee, which may make interpretation of imaging studies and radiology reports challenging for physicians.^6,8^ Similar to other musculoskeletal conditions, patient demographic characteristics and social determinants of health may influence treatment decisions. Given the high variability between practice patterns and recommendations regarding meniscus surgery, this condition has significant potential for influence by social factors, such as race, ethnicity, and insurance status. The effect of these factors on patient care decisions, however, is currently unclear.

The widespread use of electronic health records allows for large collections of data. Machine learning algorithms, including natural language processing (NLP), have shown promise in allowing for large-scale evaluations of patient data.^21^ Leveraging population-level data may allow for better delineation of the factors that drive treatment directions for these patients.

The purpose of this study was to investigate whether specific findings from MRI reports can predict eventual treatment for patients with meniscus tears on MRI. We hypothesized that tear pattern features, abnormalities through the rest of the knee joint, and social factors would be significantly predictive of both operative and nonoperative treatment pathways.

## Methods

### Overview

This is a retrospective review of data collected through the Information Commons at the University of California, San Francisco, a data warehouse derived from prospectively collected data in the electronic health record from 2011 to 2022, which includes ~5 million patients. Deidentified patient data, including demographics, insurance payor type, and MRI reports, were collected. Demographic data included age, patient sex, race, and ethnicity. Different interventions prescribed to patients were similarly recorded.

### Cohort Identification

Patients were included in this study if they were diagnosed with International Classification of Diseases, 9th Revision and 10th Revision (ICD-10) codes associated with a meniscus tear. Patients were also included if they had Current Procedural Terminology codes for MRI of the lower extremity, meniscectomy, meniscus repair, or meniscus transplant surgery. MRI reports were filtered using Structured Query Language to only include patients with identified meniscus tears. Patients were excluded if undergoing imaging after meniscus surgery or if they had an associated ligament injury.

Patients were classified according to the 1 or multiple treatments they received. Treatments rendered included physical therapy (PT) referral, a prescription for nonsteroidal anti-inflammatory drugs (NSAIDs), opioid medication prescription, an intra-articular injection, or surgical treatment. Surgical treatment was classified as meniscectomy or meniscus repair.

### Named Entity Recognition and the Clinical Text Analysis and Knowledge Extraction System

To extract relevant data from MRI reports, we used the clinical Text Analysis and Knowledge Extraction System (cTAKES), an open-source NLP system developed by the Mayo Clinic in 2010 that is compliant with the Health Insurance Portability and Accountability Act of 1996.^22^ cTAKES uses proprietary linguistic identification algorithms in conjunction with the Unified Medical Language System, a library of standardized and comprehensive biomedical terms, to identify specific information from clinical reports. The performance of cTAKES has been validated and has outperformed similar systems when extracting particular constructs from clinical reports.^22,23^

A custom vocabulary of medical terms related to meniscus tears was provided to cTAKES. This vocabulary was developed through chart review and the recording of the different ways radiologists described various tear patterns and other pertinent radiographic findings in 1000 random MRI reports. Postprocessing of cTAKES identification was performed using Structured Query Language within Microsoft Access (Microsoft Corporation). Accuracy of identification was assessed by choosing 100 randomly classified reports and validating the appropriate classification by chart review. Sensitivities and specificities of particular entities present in these reports were also calculated.

### Statistical Analysis

Statistical analysis was performed with Stata 16.1 (StataCorp). Summative data were determined for demographic variables. Proportions were calculated for descriptions of tear types and other findings at the knee joint from the radiology report, as well as the proportion of patients receiving different treatment modalities. The proportion of patients undergoing surgery in the short term (within 90 days of MRI report) and at any time was determined. Multivariate logistic regression modeling was performed to construct models of predicting the reception of different treatment modalities as a function of demographic factors, tear characteristics, and other findings on the knee MRI report. Significance was defined as *P* < .05.

## Results

There were 4013 patients included with an available MRI demonstrating a meniscus tear (Table 1). The mean (SD) age of the cohort was 46.1 (14.0) years, with a mean (SD) body mass index of 26.9 (5.45) kg/m^2^. The cohort was composed of slightly more male (52.6%) than female (47.4%) patients. Most patients had private commercial insurance (53.1%), and most patients were White (59.0%).

**Table 1:** Demographic Information*^*a*^*

Characteristic	Value
Age, mean (SD), y	46.08 (13.96)
Body mass index, mean (SD), kg/m^2^	26.88 (5.45)
Sex	
Female	1899 (47.4)
Male	2110 (52.6)
Race	
Asian	478 (11.9)
Black/African American	266 (6.6)
Latino	371 (9.2)
Multirace	92 (2.3)
Native American or Alaska Native	12 (0.3)
Native Hawaiian or Other Pacific Island	24 (0.6)
Other	211 (5.3)
Southwest Asian/North African	41 (1.0)
Unknown/declined	149 (3.7)
White	2369 (59.0)
Ethnicity	
Not Hispanic or Latino	3345 (83.4)
Hispanic or Latino	361 (9.0)
Unknown/declined	307 (7.7)
Insurance type	
Commercial	2130 (53.1)
Medicare	364 (9.1)
Medicaid	338 (8.4)
Health maintenance organization	311 (7.8)
State-run exchange insurance (Covered California)	114 (2.8)
Workers’ compensation	17 (0.4)
Other/self-pay	739 (18.4)

a Values are presented as number (%) unless otherwise indicated.

Combined tears of both the medial and lateral menisci (47.4%) were most common, followed by isolated medial meniscus tears (37.3%) and isolated lateral meniscus tears (15.3%) (Table 2). The most common tear type was a complex tear (42.3%), followed by horizontal (31.8%) and radial (29.7%). The most common associated finding on the MRI report was edema, noted on 74.9% of scans.

**Table 2:** Tear Characteristics*^*a*^*

Characteristic	No. (%)
Meniscus involvement	
Medial meniscus	1496 (37.3)
Lateral meniscus	613 (15.3)
Both medial and lateral	1904 (47.4)
Meniscus tear descriptors	
Complex	1696 (42.3)
Flap	648 (16.2)
Bucket handle	232 (5.8)
Horizontal	1275 (31.8)
Oblique	1132 (28.2)
Radial	1190 (29.7)
Root	959 (23.9)
Vertical	357 (8.9)
Other knee findings on MRI report	
Edema	3006 (74.9)
Chondral loss	2549 (63.5)
Cystic changes	2124 (52.9)
Fissure	2006 (50.0)
Full-thickness cartilage loss	1906 (47.5)
Bone marrow edema	1822 (45.4)
Synovitis	1223 (30.5)
Osteophyte	887 (22.1)
Tendinosis	705 (17.6)
Mucoid degeneration	667 (16.6)
Parameniscal cyst	536 (13.4)
Tricompartmental degeneration	328 (8.2)
Loose body	291 (7.3)
Arthrosis	155 (3.9)

a MRI, magnetic resonance imaging.

**Figure 1.**
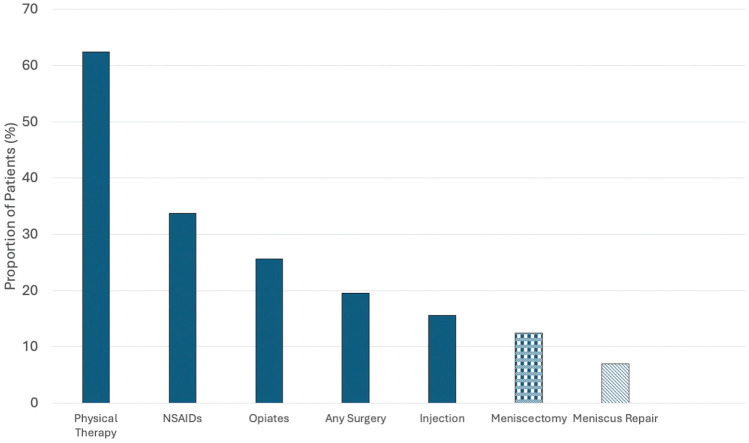
The proportion of patients receiving each treatment type is displayed for this cohort of 4013 patients with meniscus tears on magnetic resonance imaging scans. Physical therapy was the most commonly prescribed treatment modality. Any surgery includes meniscectomy and meniscus repair.

PT was the most commonly prescribed treatment (62.4% of patients; Figure 1). Specific tear patterns, including vertical tears (odds ratio [OR], 1.75), radial tears (OR, 1.48), horizontal tears (1.37), longitudinal tears (OR, 1.30), root tears (OR, 1.26), and complex tears (OR, 1.22), were associated with receiving a PT prescription (Table 3). Patients with Medicaid insurance (OR, 1.47), those with health maintenance organization (HMO) insurance (OR, 1.42), and female patients (OR, 1.25) were more likely to receive a PT prescription. Patients who had tricompartmental degenerative changes (OR, 0.70), had other insurance types (OR, 0.18), and were younger (OR, 0.98) were less likely to be prescribed PT.

**Table 3:** Multivariate Analyses for Independent Predictors of Receiving Nonsurgical Treatment Modalities*^*a*^*

Characteristic	Positive Predictors	Odds Ratio	95% CI	*P* Value	Negative Predictors	Odds Ratio	95% CI	*P* Value
Received a prescription for physical therapy	Vertical tear	1.75	1.28-2.39	<.001	Other/self-pay insurance	0.18	0.14-0.22	<.001
	Radial tear	1.48	1.24-1.76	<.001	Tricompartmental degeneration	0.70	0.52-0.94	.017
	Medicaid insurance	1.47	1.07-2.01	.018				
	Loose body	1.43	1.04-1.97	.03	Age	0.98	0.98-0.99	<.001
	HMO	1.42	1.06-1.91	.02				
	Horizontal tear	1.37	1.1-1.7	.005				
	Longitudinal tear	1.30	1.02-1.66	.034				
	Root tear	1.26	1.04-1.52	.019				
	Female sex	1.25	1.06-1.47	.007				
	Complex tear	1.22	1.02-1.45	.026				
Received a prescription for opioid medication	Vertical tear	1.75	1.36-2.26	<.001	Other/self-pay insurance	0.53	0.4-0.71	<.001
	Medicare insurance	1.69	1.17-2.17	.003	Horizontal tear	0.78	0.62-0.97	.025
	Arthrosis	1.62	1.08-2.42	.019	Parameniscal cyst	0.78	0.61-0.998	.048
	Medial and lateral meniscus involved	1.48	1.24-1.77	<.001	Age	0.97	0.96-0.97	<.001
	Medicaid insurance	1.34	1.02-1.77	.038				
	Bone marrow edema	1.29	1.07-1.54	.007				
	Posterior horn tear	1.26	1.02-1.57	.035				
	Increased BMI	1.05	1.03-1.06	<.001				
Received an intra-articular injection	Female sex	1.9	1.55-2.33	<.001	Bucket-handle tear	0.33	0.16-0.67	.002
	Medicaid insurance	1.54	1.1-2.15	.012	Other/self-pay insurance	0.35	0.23-0.52	<.001
	Chondral loss	1.51	1.17-1.95	.001	HMO insurance	0.67	0.47-0.96	.028
	Increased BMI	1.05	1.03-1.07	<.001	Posterior horn tear	0.76	0.59-0.99	.049
	Older age	1.04	1.03-1.06	<.001				
Received a prescription for NSAIDs	HMO insurance	1.54	1.19-1.98	.001	Other/self-pay insurance	0.47	0.36-0.61	<.001
	Other race	1.50	1.09-2.06	.012	Parameniscal cyst	0.75	0.60-0.93	.01
	Medicaid insurance	1.41	1.08-1.83	.011	Younger age	0.97	0.96-0.98	<.001
	Vertical tear	1.33	1.04-1.70	.024				
	Synovitis	1.26	1.07-1.48	.024				
	Edema	1.22	1.00-1.48	.048				
	Bone marrow edema	1.21	1.03-1.43	.024				

a BMI, body mass index; HMO, health maintenance organization; NSAID, nonsteroidal anti-inflammatory drug.

The most common medical management was NSAIDs, prescribed for 33.8% of patients. Positive predictors included patients with vertical tears (OR, 1.33), synovitis (OR, 1.26), edema (OR, 1.22), and bone marrow edema (OR, 1.21). Insurance status was also a predictor of NSAID prescription, with HMO insurance (OR, 1.54) and Medicaid insurance (OR, 1.41) as positive predictors. Patients who had parameniscal cysts (OR, 0.75), were younger (OR, 0.97), and had other insurance types (OR, 0.47) were less likely to receive NSAIDs.

Opioid medications were prescribed for 25.6% of patients. Knee-specific findings that were positive independent predictors of receiving an opioid prescription included a vertical tear (OR, 1.75), arthrosis/osteoarthritis (OR, 1.62), medial and lateral meniscus involvement (OR, 1.48), bone marrow edema (OR, 1.29), and posterior horn involvement (OR, 1.26). Medicare (OR, 1.69) and Medicaid (OR, 1.34) insurance were both significant positive predictors for receiving an opioid prescription, as was a higher body mass index (OR, 1.05). Negative predictors included horizontal tearing (OR, 0.78), parameniscal cysts (OR, 0.78), younger age (OR, 0.97), and other insurance (OR, 0.53).

Intra-articular injections were the least commonly administered treatment at 15.6% of patients. Injections were more commonly received by female patients (OR, 1.90), older patients (OR, 1.04), patients with a higher body mass index (OR, 1.05), patients with chondral loss (OR, 1.51), and those with Medicaid insurance (OR, 1.54). Negative predictors of receiving an injection included bucket-handle tears (OR, 0.33), HMO insurance (OR, 0.67), and posterior horn involvement (OR, 0.76), along with other insurance (OR, 0.35).

**Figure 2.**
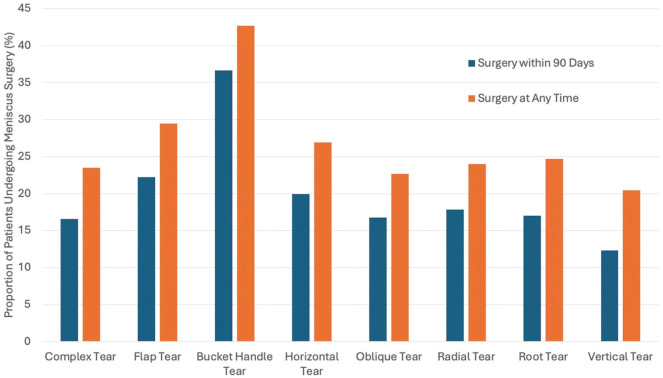
The proportion of patients undergoing surgical treatment is shown based on the meniscus tear pattern description from the magnetic resonance imaging report. Bucket-handle tears were the type most likely to undergo surgical treatment in this patient cohort.

Surgical treatment was performed for 19.6% of patients, with more patients undergoing meniscectomy (12.5%) rather than meniscus repair (7%). PT was prescribed for 90.2% of patients who eventually had surgery. There were 30.2% of patients with surgical treatment who received NSAIDs, 22.4% who received opioids, and 8.5% who received an intra-articular injection. Surgery was most commonly performed soon after the MRI was completed, with 44.2% having surgery within 30 days of the MRI and 35.6% of surgeries occurring between 30 and 90 days of the MRI. Bucket-handle tears were the most common tear type treated surgically, within 90 days of the MRI scan and also at any time after the MRI was completed (Figure 2). A bucket-handle tear was also the strongest independent predictor of surgery within 90 days of the MRI by multivariate analysis (OR, 4.04) (Table 4). Patients having surgery were significantly younger than those who did not have surgery (44.6 ± 12.7 years vs 46.4 ± 14.2 years; *P* = .001). Other positive predictors included multiracial identification (OR, 1.83), parameniscal cyst (OR, 1.80), cystic changes (OR, 1.57), flap tear (OR, 1.51), body tear (OR, 1.45), root tear (OR, 1.36), fissuring (OR, 1.33), and radial tears (OR, 1.31). A description of arthrosis/osteoarthritis was the strongest negative predictor (OR, 0.18), followed by other insurance (OR, 0.19), Medicare insurance (OR, 0.42), full-thickness cartilage defects (OR, 0.47), and medial and lateral meniscal involvement (OR, 0.62). Having surgery at any time had similar positive and negative predictors, with the addition of horizontal tears (OR, 1.32) being more likely to have surgery at any point.

**Table 4:** Multivariate Analyses for Independent Predictors of Receiving Surgical Treatment*^*a*^*

Characteristic	Positive Predictors	Odds Ratio	95% CI	*P* Value	Negative Predictors	Odds Ratio	95% CI	*P* Value
Surgery within 90 days of MRI	Bucket-handle tear	4.04	2.91-5.61	<.001	Arthrosis	0.18	0.064-0.49	.001
	Multirace/ethnicity	1.83	1.05-3.19	.033	Other/self-pay insurance	0.19	0.11-0.33	<.001
	Parameniscal cyst	1.80	1.40-2.31	<.001	Medicare insurance	0.42	0.27-0.65	<.001
	Cystic changes	1.57	1.07-2.31	.02	Full-thickness cartilage loss	0.47	0.32-0.69	<.001
	Flap tear	1.51	1.19-1.92	.001	Medial and lateral meniscus involved	0.62	0.50-0.76	<.001
	Body tear	1.45	1.17-1.81	.001				
	Root tear	1.36	1.09-1.69	.007				
	Fissure	1.33	1.09-1.63	.006				
	Radial tear	1.31	1.07-1.61	.01				
Surgery at any time	Bucket-handle tear	3.42	2.49-4.70	<.001	Other/self-pay insurance	0.19	0.12-0.30	<.001
	Parameniscal cyst	1.70	1.34-2.15	<.001	Arthrosis	0.24	0.11-0.52	<.001
	Flap tear	1.52	1.21-1.90	<.001	Medicare insurance	0.39	0.26-0.59	<.001
	Cystic changes	1.47	1.02-2.11	.04	Full-thickness cartilage loss	0.51	0.35-0.73	<.001
	Body tear	1.45	1.18-1.77	<.001	Medial and lateral meniscus involved	0.66	0.55-0.80	<.001
	Root tear	1.41	1.15-1.73	.001				
	Horizontal tear	1.32	1.04-1.66	.02				
	Radial tear	1.21	1.00-1.47	.049				
Surgery at any time after prescription of physical therapy	Bucket-handle tear	2.71	1.89-3.90	<.001	Arthrosis	0.22	0.093-0.53	.001
	Parameniscal cyst	2.03	1.55-2.65	<.001	Medicare insurance	0.29	0.19-0.46	<.001
	Discoid meniscus	1.74	0.60-0.98	.030	Other/self-pay insurance	0.46	0.27-0.78	.004
	Flap tear	1.59	1.24-2.04	<.001	Full-thickness cartilage loss	0.57	0.37-0.86	.008
	Body tear	1.45	1.16-1.81	.001	Medial and lateral meniscus involved	0.60	0.49-0.74	<.001
	Root tear	1.31	1.05-1.64	.019	Tricompartmental degeneration	0.62	0.38-1.00	.05
					Female sex	0.80	0.66-0.98	.03

a MRI, magnetic resonance imaging.

Of the patients who were prescribed PT (n = 2504), 710 patients (28.4%) had eventual meniscus surgery. Specific tear types were identified as positive predictors of surgical treatment for this subset of patients, with bucket-handle tears, parameniscal cysts, discoid meniscus, flap tears, body tears, and root tears all significant independent predictors. Patients were less likely to have surgery after starting PT for the following conditions: arthrosis, full-thickness cartilage loss, medial and lateral involvement, tricompartmental degenerative changes, Medicare insurance, and female sex.

Patients were more likely to have a meniscectomy in the setting of a flap tear (OR, 1.95), discoid meniscus (OR, 1.81), bucket-handle tear (OR, 1.61), meniscal body tear (OR, 1.55), parameniscal cyst (OR, 1.54), horizontal tear (OR, 1.40), or complex tear (OR, 1.36). Increasing age was associated with meniscectomy (OR, 1.02), along with fissuring (OR, 1.31) (Table 5). Negative predictors for meniscectomy included arthrosis (OR, 0.32), Medicare insurance (0.32), full-thickness cartilage defects (OR, 0.47), female sex (OR, 0.76), edema (OR, 0.77), and extrusion (OR, 0.77).

**Table 5:** Multivariate Analysis for Independent Predictors of Meniscectomy and Repair Surgery

Characteristic	Positive Predictors	Odds Ratio	95% CI	*P* Value	Negative Predictors	Odds Ratio	95% CI	*P* Value
Undergoing meniscectomy surgery	Flap tear	1.95	1.53-2.49	<.001	Other/self-pay insurance	0.22	0.13-0.38	<.001
	Discoid meniscus	1.81	1.09-3.03	.023	Arthrosis	0.32	0.12-0.81	.016
	Bucket-handle tear	1.61	1.09-2.39	.017	Medicare insurance	0.32	0.20-0.52	<.001
	Body tear	1.55	1.21-1.99	<.001	Full-thickness cartilage loss	0.47	0.31-0.71	<.001
	Parameniscal cyst	1.54	1.17-2.02	.002				
	Horizontal tear	1.40	1.07-1.83	.014	Female sex	0.76	0.62-0.94	.013
	Complex tear	1.36	1.08-1.70	.008	Edema	0.77	0.60-0.99	.046
	Fissure	1.31	1.05-1.63	.018	Extrusion	0.77	0.61-0.98	.030
	Increased age	1.02	1.01-1.03	<.001				
Undergoing meniscus repair surgery	Bucket-handle tear	4.60	3.13-6.75	<.001	Arthrosis	0.17	0.039-0.71	.015
	Root tear	2.31	1.73-3.09	<.001	Other/self-pay insurance	0.19	0.083-0.44	<.001
	Parameniscal cyst	1.64	1.15-2.34	.006	Medial and lateral meniscus involved	0.55	0.41-0.74	<.001
					Complex tear	0.72	0.54-0.97	.032
					Younger age	0.97	0.96-0.99	<.001

Meniscus repair was most strongly predicted by bucket-handle tear (OR, 4.6) and meniscus root tear (OR, 2.31). Parameniscal cyst was also associated with a greater likelihood of meniscus repair (OR, 1.64). Patients were less likely to have a meniscus repair with arthrosis (OR, 0.17), medial and lateral involvement (OR, 0.55), complex tear pattern (OR, 0.72), and increasing age (OR, 0.97).

## Discussion

In this large cohort of patients with MRI reports indicating the presence of a meniscus tear, we observed that specific descriptions in the radiology report of the meniscus tear pattern and the whole knee joint, in addition to demographic characteristics, are associated with the prescribed treatment. We observed significant variability in the treatment provided to patients with meniscus tears. In these MRI reports, meniscus tears were present with numerous other findings throughout the knee, and these coexisting pathologies are important in determining which treatment patients receive.

The combination of specific language from a radiology report and the use of big data and advanced analysis tools shows the potential for leveraging available electronic medical record data to improve the understanding of current management patterns of common health care conditions. Decision support tools may have the potential to aid physicians in interpreting diagnostic studies.^10,26^ This support may be useful for internists, family physicians, and other nonspecialists who are faced with interpreting complex imaging reports and attempting to initiate treatment. Specific tears, such as bucket-handle tears and root tears, would benefit from earlier surgical referral, while descriptions of arthrosis, medial and lateral meniscal involvement, and full-thickness cartilage loss may suggest that surgical referral is not immediately necessary. With patients having increased direct access to imaging reports, these findings could also help in developing patient-facing tools to translate the medical language of a radiology report into direct interpretations. Importantly, these findings must be interpreted with attention to a patient's symptoms, examination findings, and treatment goals, although providing guidance in interpreting these findings could help all physicians better understand the indicated treatment options. It is also essential to realize that current treatment patterns are not necessarily best practice, and careful attention must be given to defining personalized protocols that provide optimal outcomes. Future developments in artificial intelligence are likely to offer even further advancements in defining patient-specific factors that can better predict individualized benefits from specific treatments.

Physical therapy was the most commonly prescribed treatment modality, which is in line with most recommendations regarding patients with meniscus tears.^11,12,19^ In patients who were initially treated with PT, bucket-handle tears, discoid meniscus, flap tears, body tears, and root tears were identified as more likely to have eventual surgery. This observation suggests that these tear types may benefit from earlier surgical evaluation. It is clear that these tear subtypes must be identified clearly, which is a current shortcoming of the ICD-10 coding of meniscus tears. Many of these tear types can be categorized as “complex,”“peripheral,” or “other” within the ICD-10 system, which limits the ability of future studies on administrative claims data. The current study also shows high rates of prescribing formal PT. The effectiveness of a formal program compared with self-directed home PT could be important to determine in defining the best treatment directions for patients with meniscus tears.

Opioid medications were prescribed to a surprisingly high proportion of the patients in this cohort. The overprescription of opioid medication is an epidemic in the US health care system.^22,23^ Opioid prescription rates of greater than 25% of patients in this cohort highlight the extreme societal impact that health care providers have in prescribing these medications in common musculoskeletal conditions. Many of the positive predictors of receiving opioid prescriptions identified in this cohort are in direct opposition to the predictors observed in the patients undergoing surgical treatment. This use of narcotic pain medication may be prompted by the limited treatment options for early symptomatic arthritis, highlighting this treatment gap again as one that is in need of novel therapeutics.^17^ Previous work has also shown that nonnarcotic medications, such as acetaminophen, NSAIDs, and intra-articular injections, are often as effective as opioids in the management of musculoskeletal pain.^20^ Decreasing opioid prescribing in a condition as common as meniscus tears could have a large impact on the overall overutilization of narcotic medication.

These findings should be interpreted with a clear understanding of the limitations of the study. First, we sought to evaluate the accuracy of the NLP methods and showed a high degree of accuracy, although there will still be some likely misclassifications within the data set. There may also be meniscus tears that were misread or incorrectly described by the evaluating radiologist. There may be variability in the quality of the radiology interpretation or variations in scan quality that can affect interpretation. We are not able to include the expertise or training background of each radiologist. Similarly, we do not have information on the training and experience of the treating provider, which likely affects treatment decisions. Many other factors not evaluated in our cohort can also contribute to eventual treatment, including the preferences of the treating provider, manifestation of symptoms, physical examination findings, injury mechanism, and the specific goals of the patient. This current study reflects the current patterns for treating patients with meniscus tears on MRI but may not necessarily be the best treatment plan. For many patients, imaging may not be needed for treatment decisions, and there may also be a population of patients with meniscus tears who have not undergone advanced imaging and have received different treatment plans. While our sample size is large and represents a diverse patient population in a major metropolitan area, the findings should be examined in different geographical and cultural areas with potentially different practice patterns. We are also unable to track compliance with the treatment prescribed. There is also a possibility of patients receiving additional treatment outside of what was prescribed or outside of our health care system. We also did not evaluate knee replacement as a surgical outcome, although this may be an eventual treatment procedure for some patients. We do not have data on displaced meniscus tears or potential locked knees or restrictions in motion, which often drive treatment decisions. A final limitation is the lack of any patient-reported outcome measures. We describe only the treatment received but not the beneficial or negative effects that any treatment can have. Future studies that incorporate a response to treatment would be essential in understanding the clinical implications of these findings.

In conclusion, we have observed that specific meniscus tear types and other findings at the knee joint on MRI, as well as demographic variables, are significantly associated with treatment for meniscus tears. Bucket-handle and root tears were most likely to undergo surgical treatment, while patients on Medicare and those with arthrosis were more likely to be treated with nonoperative modalities. Prescription of opioid medications in meniscal tears was observed at a higher-than-expected rate, and this finding should prompt attention toward both minimizing opioid prescribing in this patient population and developing alternative treatment pathways for these patients. This study highlights that NLP of big data, including radiology findings, may have a role in predicting eventual treatment for this common finding and guiding therapeutic guidelines, although these treatment decisions still must be made on a patient-specific basis.
